# Efficacy of Intravenous Acetaminophen in Length of Stay and Postoperative Pain Control in Laparoscopic Roux-en-Y Gastric Bypass Surgery Patients

**DOI:** 10.1089/bari.2018.0005

**Published:** 2018-09-01

**Authors:** Matthew Lange, Christina W. Lee, Tara Knisely, Subbaiah Perla, Kimberly Barber, Michael Kia

**Affiliations:** ^1^ Department of Surgery, Genesys Regional Medical Center, Grand Blanc, Michigan.; ^2^ Department of Surgery, University of Wisconsin School of Medicine and Public Health, Madison, Wisconsin.; ^3^ Office of Research, Genesys Regional Medical Center, Grand Blanc, Michigan.; ^4^ Department of Mathematics and Statistics, Oakland University, Rochester, Michigan.; ^5^ Department of Surgery, McLaren Regional Medical Center, Flint, Michigan.

**Keywords:** pain, laparoscopic Roux en Y gastric bypass, intravenous acetaminophen, length of stay, postoperative pain control, bariatric

## Abstract

***Background:*** Opiate-based pain medications may incur adverse effects following bariatric surgery. The aim of this study was to evaluate the efficacy of intravenous Acetaminophen (IVAPAP) on length of stay (LOS) after laparoscopic Roux-en-Y gastric bypass (LRYGB) surgery.

***Methods:*** This was a prospective, double-blind, randomized controlled trial conducted from October 2011 to March 2014 at a 416-bed teaching hospital. Eighty-nine total patients were included (control group, *n* = 45; treatment group, *n* = 44). Patients were administered either 1000 mg of IVAPAP or placebo every 6 h beginning preoperatively and continuing for four doses. LOS, total narcotic consumption, pain and nausea scores, time to return of flatus (ROF), and postoperative rescue pain medication used were measured during the first 24 h after surgery.

***Results:*** LOS was significantly decreased in the treatment group compared with control (2.72 days vs. 3.18 days; *p* = 0.03). There was significant reduction in time to ROF (1.87 days vs. 2.24 days; *p* = 0.04). Pain was significantly decreased in the first 2 postoperative hours in the treatment group (*p* = 0.02). Total opioid consumption, postoperative nausea scores, and use of rescue pain medications were not affected.

***Conclusions:*** The use of IVAPAP significantly decreases LOS following LRYGB, improves acute postoperative pain control, and mediates quicker return of bowel function.

## Introduction

Postoperative pain remains an unavoidable consequence of bariatric surgery.^1^ Traditional narcotic-based pain managements have been reported to have multiple adverse effects. The most frequently cited narcotic-related adverse effects include nausea and vomiting, urinary retention, fatigue, pruritus, dizziness, headaches, ileus, and respiratory failure.^2–4^ Previous studies have identified associations between opioid consumption and increased length of stay (LOS) following laparoscopic Roux-en-Y gastric bypass (LRYGB).^5^ As a result, nonnarcotic analgesics have garnered interest as potential useful adjuncts in the postoperative setting.

In many countries, intravenous Acetaminophen (IVPAP) is a commonly used pain medication and serves as an adjunct to the use of opioid pain medications. In the US, it has only recently been studied in a variety of surgical patients and deemed an appropriate adjunct to standard postoperative opioid agents in multimodal analgesic models.^5–17^ These studies have shown that the use of IVAPAP decreases the overall need for opioids, minimizes adverse effects, and decreases LOS.^7–9^ In the bariatric patient population, there are limited retrospective data on the benefits of IVAPAP in combined multimodal therapy. Therefore, the purpose of this prospective study was to evaluate the effect of IVAPAP in bariatric patients who undergo LRYBGP with respect to overall hospital LOS as well as opiate-induced adverse events. The primary hypothesis was that by administering IVAPAP to our LRYGB patients, we would see an overall shorter LOS in the hospital as well as decreased use of postoperative opioid medications.

## Methods

### Trial design

This study was a single-institution, prospective, randomized, double-blinded placebo controlled trial conducted at a community teaching hospital between October 2011 to March 2014. The study was approved by the hospital's Institutional Review Board and was registered at ClinicalTrials.gov (NCT01460667). All patients provided written preoperative consent to participate in the study before enrollment. All patients underwent surgery by a single supervising surgeon.

### Subjects

A total of 110 patients were enrolled in the study. Patients were then randomized after being scheduled for LRYGB. This study included male and female adults between the ages of 18 and 65 years, with a body mass index (BMI) greater than 35 kg/m^2^ and American Society of Anesthesiologists (ASA) score 1, 2, or 3. Exclusion criteria consisted of hypersensitivity to acetaminophen, use of opiate medications before the study for greater than 7 days, or hepatic insufficiency that would preclude the use of acetaminophen. Discharge criteria included tolerance of bariatric diet, oral medication pain control, and return of bowel function.

### Materials and randomization

Pharmacy personnel were instructed and trained before the start of the study and responsible for the randomization process on the day of surgery. One-hundred milliliters of normal saline (NS) placebo or IVAPAP* (Ofirmev™) was prepared and blinded by pharmacy. All active drug and placebo solutions, containers, and labels were identical in appearance, avoiding any recognizable symbols. Funding from Mallinckrodt Pharmaceuticals (St. Louis, MO) was used to cover the cost of the IVAPAP provided in this study.

#### Study groups

The control group received standard postoperative analgesia supplemented with four scheduled doses of NS as placebo. The treatment group received four scheduled doses of 1000 mg IVAPAP. All study medications were prepared by a designated pharmacist allocated to the study in identical unlabeled bottles on the day of surgery. Each group received the first dose of the placebo or the study medication preoperatively and continued for a total of four doses at 6 h intervals.

All patients were allotted hydromorphone via patient-controlled analgesia (PCA) (0.1 mg with an 8-min lock-out). Each patient had an OnQ** (Halyard Health) pain system placed at the conclusion of surgery that stayed in place until discharge or for a total of 5 days, if the patient remained in the hospital. The OnQ Pain Relief System* is a nonnarcotic elastomeric pump (400 mL ball) that delivers (4 mL/h) a flow of local anesthetic (0.5% Bupivacaine) to the patient's surgical site through two copper catheters placed in the operating room. Narcotic consumption per patient was recorded by nursing staff over the initial 24 postoperative hours with time “zero” and PCA initiation starting upon arrival to the floor from postanesthesia care unit (PACU).

#### Pain and nausea

A visual analog pain scale (VAS, scale 0–100) and nausea scoring (0–100) was measured at 2 h intervals postoperatively once the patients were admitted to the floor (time “zero”) from the PACU. Pain levels were assessed using the VAS system from 0 to 100 with 0 representing no pain and 100 representing the most severe pain. Pain and nausea levels were assessed by nursing staff and entered into the electronic medical record. The assessment of pain scores during a subject's sleep was evaluated using the last-observation-carried-forward method.

#### Rescue medications

Patients experiencing breakthrough pain received IV Hydromorphone or Fentanyl depending upon the level of pain. Study personnel were instructed to offer rescue medication to patients who reported pain intensities at 80 or greater on the VAS. Rescue medications were IV Hydromorphone unless the patient had an adverse reaction to this medication at which time alternative IV opioid medication (Fentanyl) was administered. After the initial 24 h, the choice of rescue medications was expanded to include Ketorolac. Consumption of rescue medications was converted to an oral morphine-equivalent dose for analysis using a prespecified conversion factor (Table 1).^18^ All study medications were stopped after 24 h, which comprised the data collection period of the study.

**Table 1.** Dose Equivalents for Opioid Analgesics to Oral Morphine Equivalent

*Opioid analgesic*	*Parenteral dose*	*Oral morphine equivalent*
Intravenous hydromorphone	1.5 mg	30
Intravenous fentanyl	100 mcg	30

#### Data collection

Clinical characteristics (BMI, age, and gender), surgical procedure, narcotic consumption (mg), postoperative pain and nausea score, return of bowel function by means of passing flatus, date of discharge, LOS, and the number of pain rescue medications were assessed and analyzed.

#### Clinical and safety endpoints

Study primary endpoint was LOS, while secondary endpoints were postoperative PCA opioid usage (mg), return of flatus (ROF), pain (VAS) scores, nausea scores, and rescue pain medication usage (number of times needed) in the initial 24-h postoperative time period. Adverse events were defined as any complication or unwanted event experienced by the patient in association with the study medication. All patients underwent preoperative screening, including complete blood count and liver function tests (aspartate aminotransferase, alanine aminotransferase, and Total Bilirubin). Women of reproductive age (18–55) completed a urine pregnancy test preoperatively.

### Statistical analyses

LOS, time to ROF, opioid consumption and postoperative pain scores were compared for the control and treatment groups using a two-sample *t*-Test. The Wilcoxon Rank-sum test was used for comparison in LOS and time to ROF. The postoperative pain scores were analyzed using a repeated measures analysis of variance. Rates and comparison of rescue pain medications given were analyzed using the chi-square test. This study was powered to detect differences between the two groups in LOS and narcotic use of 20% or greater at a beta power level of 0.85 and a significance of *p*-value <0.05. At least 110 subjects (55 each group) was required to achieve this calculated power. Statistical analysis was carried out using MINITAB 17 Statistical Software (2010). Statistical significance was denoted by *p*-value <0.05.

## Results

**FIG. 1.**
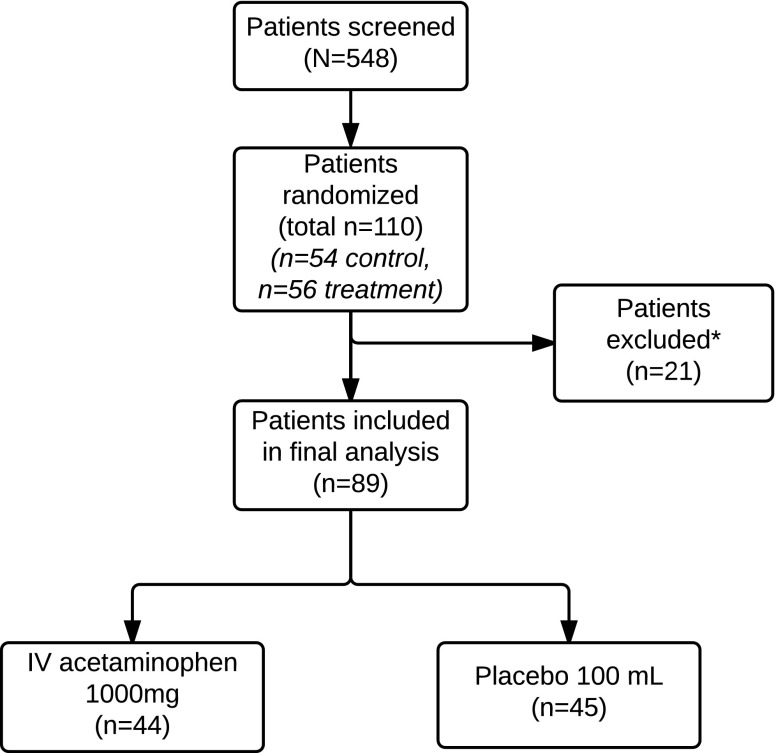
Flow of patients through a randomized, double-blind, placebo-controlled, single-center study of an intravenous acetaminophen dosing regimen versus placebo for the treatment of pain after laparoscopic Roux-en-Y bariatric surgery. *Reasons for exclusion: insufficient data, patient declined after initial consent, five underwent different procedure, and one had an allergic reaction.

A total of 110 patients who met criteria for our study were selected and randomized into either the treatment group or the control group. Of the initial 110 participants, 54 were in the control group and 56 in the treatment group. Eighty-nine were included in the final data analysis (Fig. 1). Among the 21 (19%) patients who were excluded in analysis, 11 patients (6 from the treatment group and 5 from the placebo group) had no data collected postoperatively in terms of pain score data as well as PCA narcotic usage or declined to participate after initially providing informed consent. Five patients underwent surgery other than the intended LRYGB, secondary to intraoperative findings, four of the cases were cancelled preoperatively by anesthesia and one patient developed an allergic reaction to the study drug.

Among the 89 patients included in the final analysis, 68 (76%) were female and 21 (24%) were male (*p* = 0.8). The mean age was 45.3 years in the control group compared with 43.6 years in the treatment group (*p* = 0.48). The mean BMI of the 89 patients was 49.1 kg/m^2^ in the control group and 50.9 kg/m^2^ in the treatment group. There were no significant differences between the groups in regard to demographic variables (gender, age, and BMI).

### Primary outcome

#### Length of stay

The LOS was recorded from the time of admission on the day of the operation, considered day 0 until the time of discharge. The LOS ranged from 2 to 7 days with a median of 3 (variance = 1.5) days in the control group and a median LOS of 3 days (variance = 0.39) in the treatment group with a range of 2–4 days. The Median Test for two Independent Samples showed a significant difference (*p* = 0.035) with the distribution of the treatment group being significantly fewer days than control. The addition of IVAPAP to the postoperative pain regimen led to an overall reduction in LOS of nearly half of a day (0.46 days) (*p* = 0.03). The control group did have three patients with a LOS of 6 and 7 days, while the treatment group had all LOS between 2 and 4 days.

### Secondary outcomes

#### Time to return of bowel function

Patients who received IVAPAP supplementation demonstrated an overall reduction in the time to ROF compared with control. They demonstrated a statistically significant decrease in time to return of bowel function with an average time of 1.87 days versus 2.24 days in the control group (*p* = 0.04). Every patient in our study had a documented ROF before discharge, but this was not used as a specific discharge criterion in our patients.

### Opioid consumption

Overall hydromorphone use was measured over the first 30 h postoperatively between the two groups and total narcotic use was similar between the two groups (*p* = 0.64). A total of 6.76 mg of hydromorphone was used in the initial 30-h period in the control group and 6.34 mg used in the IVAPAP group (Table 2). Hydromorphone use was also evaluated in 6 h intervals corresponding to each dose of the Ofirmev*/placebo and each 6-h grouping was found to have used a similar amount of narcotic usage over each period (Table 2). The PCA was left in place for 6 h after the last dose of the study medication was given to assess for any significant change in narcotic usage once the study medication was completed.

**Table 2.** Overall Patient-Controlled Analgesia Usage (mg) Over 30 H Period Postoperatively

	*Group*		
*PCA total (h)*	*Control*	*Treatment*	p
PCA 1–6	2.02	2.10	0.810
PCA 7–12	1.50	1.51	0.996
PCA 13–18	1.35	1.26	0.707
PCA 19–24	1.43	1.13	0.169
PCA 25–30	0.46	0.39	0.591
PCA total (1–30)	6.76	6.37	0.641

PCA, patient-controlled analgesia.

### Pain and nausea scores

**FIG. 2.**
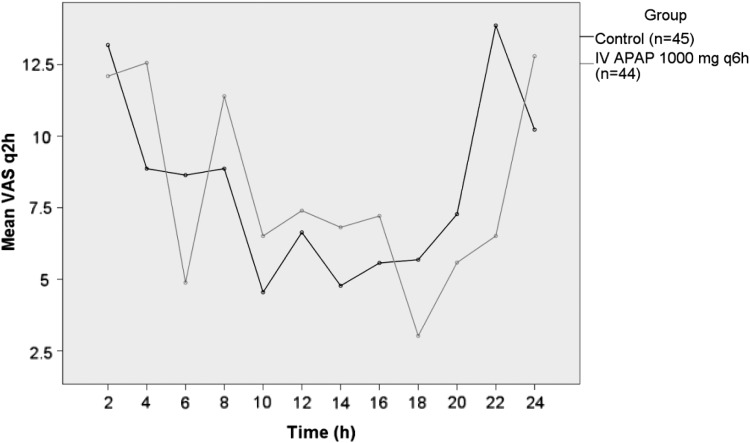
Mean scores for pain intensity, based on 100-mm visual analog scale at each 2-h interval up to 24 h. IVAPAP was given in the treatment group 1000 mg IV every 6 h versus the placebo/control group. IVAPAP, intravenous Acetaminophen.

The pain and nausea scores were measured at strict 2-h intervals on a scale of 0 to 100 using a visual analog scale for the first 24 h postoperatively. The median pain score was 25.5 (SD = 16.2) in the control group and 29.1 (SD = 16.1) in the IVAPAP group over the first 24 h postoperatively. The difference was not statistically significant (*p* = 0.29). There was no gender difference in baseline pain scores (male = 21, female = 68, *p* = 0.8). Pain improvement (as shown by a decreased score) was analyzed initially (2 h postoperatively) and latently (beyond 2 h). A significant decrease was observed for the initial period at the initial 2-h postoperative pain check (IVAPAP group = 38.8, control group = 46.7; *p* = 0.02), but no further change was observed for the latent period (IVAPAP group = 25.5, control group = 29.1; *p* = 0.79) (Fig. 2). The severity of nausea was the same in both groups. The median postoperative nausea scores were similar: 8.4 for control and 7.5 for the IVAPAP group (*p* = 0.66).

### Rescue medications

A total of 24 (26%) patients needed rescue pain medications either IV Hydromorphone or Fentanyl in the initial 24 h postoperative period, and of those, 14 (31%) were in the IVAPAP group, and 10 (22%) were in the control group. While a numerically larger number of patients in the IVAPAP group required rescue medication in the initial 24 h postoperatively compared with the control group, it did not reach statistical significance (*p* = 0.29). The rescue medications were converted to oral morphine equivalents and the placebo group used a mean of 29.45 OME, while the IVAPAP group required a mean of 21.45 OME for rescue medications showing no statistically significant difference between the two groups (*p* = 0.64).

### Adverse events

One adverse treatment incident was reported. Generalized swelling of face and rash upon initiation of the study medication caused the patient to be removed from the study.

## Discussion

Nearly 80% of patients experience acute postoperative pain after both open and laparoscopic surgery, and 40% of these patients report under treatment of pain.^1^ Multimodal analgesic therapy has been shown to provide more efficacious postoperative pain control, decrease overall LOS and decrease overall opioid-induced complications.^5,6^ This study showed that the use of IVAPAP as part of a multimodality approach to pain control in LRYGB significantly decreased LOS. While there was no significant difference in overall postoperative opioid usage, patients receiving IVAPAP had earlier recovery of bowel function. The LOS differential is possibly related to the earlier return of bowel function, which has previously been used as an endpoint in bariatric surgery for discharge eligibility. While there is minimal evidence to support the direct correlation of IVAPAP with decreased time to ROF, it provides a potential area for future investigation. This significant decrease in LOS not only directly benefits patients but also has the potential to reduce overall hospital costs significantly due to the 0.5 day reduction in LOS. While this study was not designed to examine hospital costs, the decreased LOS and potential cost-saving implications is worth investigating further. As bariatric surgery evolves, more enhanced recovery after surgery protocols are being developed to help decrease LOS even further and are challenging our previously accepted endpoints for patient discharge. ROF over the past several years is no longer considered by many to be a part of the discharge criteria for bariatric surgery, but our findings could provide significant benefit, if found to be true in other surgical specialties.

This is the only double-blind randomized controlled trial conducted to date that has evaluated the efficacy of IVAPAP on LOS and pain control in bariatric patients undergoing a LRYGB. This trial is consistent with previous studies demonstrating the effectiveness of IVAPAP in reduction of LOS. IVAPAP was associated with a shorter average LOS by 0.4–1.5 days (an overall decrease of 18%).^12–15^ Recent studies by Bamgbade *et al.*, Shaffer *et al.*, and Hansen *et al.* revealed that bariatric patients receiving IVAPAP as part of their pain regimen demonstrated earlier ambulation postoperatively, in addition to decreased LOS.^12,14^

The benefits of oral acetaminophen, when used in conjunction with narcotic pain medications, have a limited efficacy in our postoperative patient population secondary to impaired absorption as well as altered pharmacokinetics when given in conjunction with intravenous morphine.^19,20^ Furthermore, adequate absorptive capacity of the gastrointestinal tract and the availability of an oral route for medication administration may be limited even further depending on the type of surgery performed. Further limiting this patient population is the inability to use nonsteroidal anti-inflammatory drugs, as a consistent method of pain relief in our LRYGB patients. According to the Clinical Practice Guidelines for the Perioperative Nutritional, Metabolic, and Nonsurgical Support of the Bariatric Surgery Patient, NSAIDs should be strictly avoided after bariatric surgery, because of their implication in anastomotic ulceration/perforation after LRYGB.^21^ NSAID use postoperatively has been shown, in a study by Wilson *et al.*,^22^ to significantly increase the risk of postoperative development of marginal ulcerations in RYGB patients. This further illustrates the importance of developing multimodal pain regimens combining both centrally and peripheral targeted mechanisms to decrease opioid reliance and maintain a safe and acceptable side effect profile. Our study echoed this safety profile with only one adverse event stemming from IVAPAP.

IVAPAP has been shown by studies both prospectively randomized and retrospective studies to decrease postoperative narcotic use and improve patient satisfaction.^6,11,12,14,23^ This study identified no significant difference in postoperative narcotic use or difference in VAS pain score in the immediate postoperative period, except for the first 2 to 4 h. This initial decrease in pain scores is corroborated by findings of Wininger *et al.*,^16^ which shows an initial decrease in pain scores in the 1000 mg IVAPAP patient population over the placebo group.

Both study arms demonstrated similar PCA and rescue pain medication usage. An explanation for this effect may be that overall low utilization of pain medications failed to distinguish a statistical difference as the study may not have been adequately powered for such low usage. However, we found a significant difference in postoperative VAS scores in the initial postoperative period, which agreed with the Wininger *et al.* study for initial postoperative pain evaluations.

Another explanation includes a decrease in the number of narcotics required intraoperatively and during the PACU duration of monitoring. Improved postoperative pain secondary to preoperative administration of IVAPAP has been confirmed in a recent observational study by Bamgbade *et al.* evaluating perioperative pain management among bariatric surgery patients.^12^ In this study, we did not evaluate intraoperative narcotic administration between these groups, however, several studies have shown the benefits of perioperative IVAPAP usage with a reduction in overall postoperative pain, shorter LOS, and earlier ambulation postoperatively.^10,12,17^

This study also utilized On-Q pain catheters in our multimodal pain approach. While these catheters have been shown in several studies to improve postoperative pain control on their own,^24,25^ we are seeking to further improve our postoperative pain control by also adding IVAPAP to our protocol. We wanted to see if we could further reduce our postoperative narcotic use and need for “rescue pain medications.” With our relatively low utilization of the PCA postoperatively, this could have been a reason why we saw such a small difference in narcotic utilization between the treatment group and control group. With the narcotic use among both groups being so similar, a larger sample size may be required to see if a true difference exists. Several studies, both retrospective and prospective, have shown that these catheters can lead to a decrease in overall narcotic usage and pain scores.^24,25^ No difference in nausea scores or antiemetic usage was seen during this study, which is consistent with the findings of previous studies.^24,26^

Despite the prospective, randomized nature of this study, there were some limitations. This study was limited to a single institution. A single surgeon has a particular approach that may not represent other clinical practices, and therefore, the generalizability of this one study is limited. Further limitations are that a large percentage of patients were excluded for a variety of reasons, most notably due to inadequate documentation resulting in a high degree of missing data. During the trial, the routine use of upper GI swallow studies on postoperative day 1 was discontinued and changed to a more selective approach triggered by an abnormal postoperative course, such as tachycardia or persistent nausea and vomiting. This change had no effect on our trial as both study arms had equal distribution of receiving upper GI's, and 22 patients (12 control and 10 treatment) did not undergo routine postoperative upper GI.

Future directions may examine the use of a single preoperative dose of IVAPAP compared to a complete 24-h dosing regimen looking at the same outcomes discussed here with the addition of postoperative time to ambulation. This would allow us to further confirm or refute some of the recently published data in regard to fast-track protocols in postoperative bariatric management.^12^ Additional investigation into the intraoperative usage of narcotics could give us further insight into our results. Many of the studies that have been reviewed in the bariatric literature fail to look into this fact, but there are several studies that have shown that preoperative IVAPAP usage decreases intraoperative pain medication requirements.

## Conclusion

In conclusion, a multimodal approach to pain management in our bariatric patient population has shown to be beneficial in more areas than just consumption of opioids postoperatively. Addition of IVAPAP to standard opioid PCA significantly reduced the LOS following LRYGB, while also promoting a shorter time to ROF compared to patients who received narcotic medications alone.
